# Effects of plyometric vs. strength training on strength, sprint, and functional performance in soccer players: a randomized controlled trial

**DOI:** 10.1038/s41598-023-31375-4

**Published:** 2023-03-14

**Authors:** Shahnaz Hasan

**Affiliations:** Department of Physical Therapy and Health Rehabilitation, College of Applied Medical Sciences, Majmaah University, Al Majmaah, Saudi Arabia

**Keywords:** Rehabilitation, Muscle

## Abstract

Plyometric training (PT) has been shown to have numerous benefits and few harmful effects. This study aimed to compare the effects of PT vs. strength training on muscle strength, sprint, and lower limb functional performance in soccer players. Ninety participants (mean age 22.5 years) were equally and randomly divided into three groups: a plyometric training group (PTG; n = 30), a strength training group (STG; n = 30), and a control group (CG; n = 30). In PTG: bounding, hurdling, and drop jumping exercises were performed. In STG: isometric exercises of knee extension (at 60º knee flexion), quadriceps (in supine), hip adductors (in crook lying), and straight leg raise were performed. In CG: no specific training was given. All interventions were performed for eight weeks. Isometric strength (IS), sprint (ST), and single-leg triple hop (SLTH) tests were outcome measures. The wilcoxon signed ranks test was used for with-in-group analysis, and Kruskal Wallis and Mann–Whitney u tests were used for between-group analyses. In PTG: in comparison to a baseline measurement, IS increased by 8.83% (p < 0.05), ST decreased by 20.14% (p < 0.05), and SLTH increased by 7.78% (p < 0.05). In STG: IS increased by 15.76% (p < 0.05), ST decreased by 30.26% (p < 0.05), and SLTH increased by 12.41% (p < 0.05). In CG: IS increased by 5.72% (p < 0.05), ST decreased by 15.54% (p < 0.05), and SLTH increased by 4.41% (p < 0.05). The greatest improvements were observed in STG, followed by PTG and CG. Strength training was found to be more effective than PT in improving muscle strength, sprint, and lower limb functional performance in male soccer players.

## Introduction

One of the most widely-played sports in the world is soccer^1^. Soccer players are involved in tackles, jumps, frequent changes of direction, and intense sprinting^2^. Sprinting is a part of the soccer game. Under-seventeen (U-17) players of soccer game were reported to sprint an average distance of 8448 m, 428 m at speeds of > 19.1 km/h and 501 m at speeds of 16–19 km/h, and during a game of 2 × 40 min on an area of 100 × 70 m^3^. Youth soccer players require a combination of power and strength in their lower-limb muscles to win a sprinting or jumping competition or to get the ball before the rival team player^3,4^. Conditioning and strength are not only crucial for enhancing muscle performance but for preventing injuries also^5^. One of the methods to increase strength and power is plyometric training (PT). Improvements in sprint/acceleration^6,7^, power and strength of leg muscles^8^, improved agility^9^, and running performance^6,9^ have been reported due to PT. When the actions are performed quickly, the quick eccentric muscle contraction aids in greater power and force output during the subsequent concentric contraction^10–12^.

PT has been shown to have numerous beneficial effects, such as positive effects on the functioning abilities of muscles and athletic performance^11^. In addition, some studies have demonstrated that PT could enhance neuromuscular control and biomechanical technique during high-impact tasks like landing and cutting^13–15^. The likelihood of injuries in the lower extremities in team sports may be decreased by PT^16,17^. Lastly, according to the experimental data, PT appears to elicit favorable bone^18,19^ and musculotendinous adaptation^20,21^ in addition to neuromuscular adaptation. However, according to reports, PT may also have drawbacks, especially in the beginning^12^. The delayed onset of muscle soreness (DOMS) is caused by repetitive and high-intensity eccentric contractions that occur during PT^22^. In addition, a program of eccentric exercises that is vigorous enough to elicit an adaptation response in the muscles but mild enough not to debilitate the participant seriously is recommended for someone who is not accustomed to eccentric exercises to prevent the debilitating effects of muscle damage and delayed-onset muscle soreness^23^. However, this concept is contested by other studies like a study by Loturco et al.^24^.

Quadriceps muscle strength is essential for players' sporting abilities, such as jumping, sprinting, and running in most teams or individual sports, such as football, volleyball, and netball^25^. Maximal voluntary contraction of the quadriceps (MVC) and maximum ball velocity were found to be positively correlated in some studies like that of Narici et al.^26^. In addition, elite soccer players' maximal squat strength, sprinting, and jumping was found to be positively correlated in a study by Wisloff et al.^27^. It appears that soccer players need strong quadriceps; therefore, strengthening this particular muscle could result in improvements in soccer performance.

Since PT may also have a few adverse effects on athletes, strength training of quadriceps muscle may be an excellent alternative if found equally or more effective. To the best of our knowledge, no study has compared the effects of PT vs. strength training of quadriceps muscle on muscle strength, sprint, and lower limb functional performance in soccer players. Therefore, this study was conceptualized to compare these two training programs. This study aimed to compare the effects of 8-week-long plyometric training vs. strength training on muscle strength of quadriceps, sprint, and lower limb functional performance in soccer players. The study hypothesized that there is a significant difference between plyometric and strength training in soccer players.

## Materials and methods

### Study design

A three-arm parallel group randomized controlled trial was designed to compare PT's and ST's effects on the isometric muscular strength of quadriceps (IS), sprint test (ST), and single leg triple hop test (SLTH) in male soccer players.

### Sample size calculation

Before recruiting the participants, the sample size was calculated using the software G*Power version 3.1.9.4. The effect size of 0.613 (α = 0.05, power (1 − β) = 0.95) was taken from a previous study that used plyometric training as an intervention and SLTH as an outcome measure^28^. This suggested a sample size of 74 participants; however, due to the availability of participants, a total of 90 participants were recruited into the study.

### Randomization

**Figure 1:**
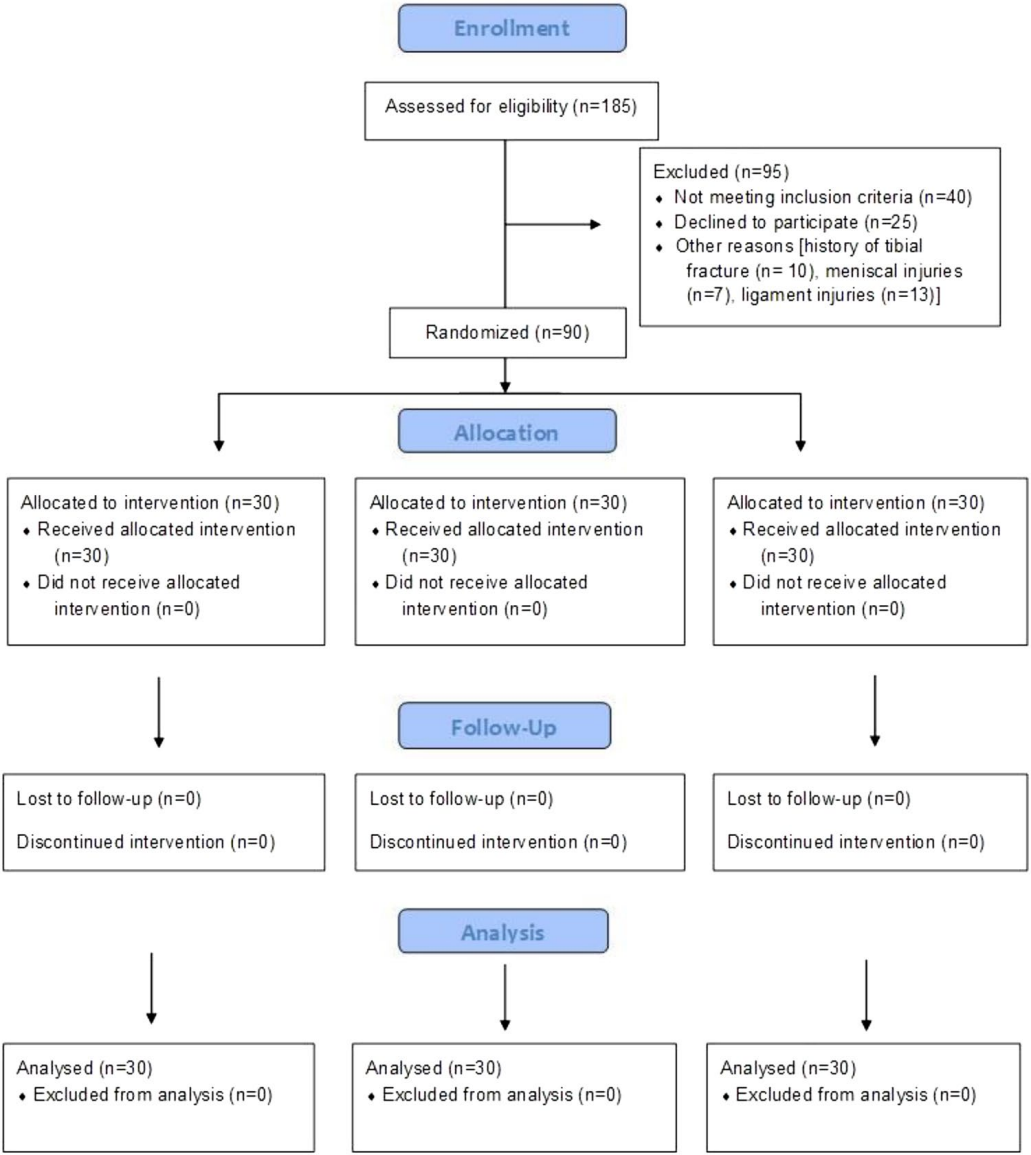
Consolidated Standards of Reporting Trials (CONSORT) flow chart showing the study's recruitment, randomization, and analysis of participants.

Ninety participants were equally and randomly divided into three groups: a Strength training group (STG; n = 30), a Plyometric training group (PTG; n = 30), and a Control group (CG; n = 30) (Table 1) (Fig. 1.). Randomization and allocation of participants to three groups were performed using the software SPSS version 26 (SPSS Inc., Chicago, IL, USA) and the lottery method. An independent researcher who was not part of this study generated the random allocation sequence, enrolled the participants, and assigned them to three groups. The outcome assessor, who was not part of the study, was blinded to the random allocation of participants.

**Table 1:** Respondent’s demographic and variables data, n = 30 in each group, mean ± SD, and p-values for Shapiro–Wilk tests of normality.

	PTG (n = 30)	p-value	STG (n = 30)	p-value	CG (n = 30)	p-value
Age (years)	22.95 ± 1.26		22.20 ± 1.84		22.36 ± 1.60	
Height (m)	1.77 ± 0.06		1.75 ± 0.05		1.73 ± 0.04	
Weight (kg)	68.96 ± 4.61		70.23 ± 2.81		69.00 ± 3.75	
BMI (kg/m^2^)	21.88 ± 1.16		22.73 ± 1.13		22.70 ± 1.23	
IS Pre (N-m)	131.70 ± 4.36	0.171	131.33 ± 5.23	0.069	130.93 ± 4.94	0.039*
IS Post (N-m)	143.33 ± 3.99		152.03 ± 3.80		138.43 ± 5.58	
ST Pre (s)	8.55 ± 0.22	p < 0.001*	8.49 ± 0.29	0.021*	8.44 ± 0.25	p < 0.001*
ST Post (s)	6.83 ± 0.38		5.92 ± 0.40		7.12 ± 0.34	
SLTH Pre (cm)	474.56 ± 11.31	0.926	475.10 ± 8.31	0.046*	475.90 ± 14.08	p < 0.001*
SLTH Post (cm)	511.53 ± 15.88		534.10 ± 18.98		496.93 ± 10.29	

*PTG* plyometric training group, *STG* strength training group, *CG* control group, *SD* standard deviation, *BMI* body mass index, *IS* isometric strength, *ST* sprint test, *SLTH* single leg triple hop test.

*Significant.

### Setting, inclusion, and exclusion criteria

The study was conducted between 30 January 2021 and 28 August 2022 at Majmaah University rehabilitation center and University Football stadium, Al Majmaah, Riyadh, Saudi Arabia. Soccer players were randomly chosen from this university and soccer clubs. Participants were screened and recruited by an experienced physiotherapist with more than 17 years of experience in assessing and managing sports and musculoskeletal disorders. The selected participants were young male athletes aged 18–25 years who participated in any level of soccer training. Participants with a cardio-respiratory disease, impairments of the spine or lower extremities, a current injury, a history of any lower limb surgery, or musculoskeletal pain as confirmed by medical and physical examinations were excluded from the study.

### Ethical approval, clinical trial registration, and informed consent

The Ethical Committee of the College of Applied Medical Science, Majmaah University, Majmaah, Saudi Arabia, approved the study (Ethics number: MUREC-Jan. 25/COM-2021/19-5). This study has been registered in a clinical trial registry (clinicaltrial.gov, ID: NCT05056792 on 27/09/2021). The study conforms to ‘The Code of Ethics of the World Medical Association (Declaration of Helsinki)’. Participants were given a detailed description of the study, its procedure, benefits, and harms before applying any intervention. All methods were performed following the relevant guidelines and regulations. All participants voluntarily gave their written informed consent.

#### Study protocol

The study was divided into three phases:

1. Pre-intervention evaluation: All participants completed three familiarization training sessions in the first week before baseline assessments. The anthropometric assessments were carried out without familiarization. Baseline data were collected before starting any intervention.

#### Outcome measures testing procedures

A. Isometric strength (IS) test: An ISOMOVE dynamometer (ISO-MANSW-IT; Tecnobody, Dalmine (BG), Italy), software version 0.0.1, was used to measure the isometric maximum peak torque of the quadriceps muscle of the dominant side. For research and clinical uses, isokinetic dynamometers offer mechanically reliable and valid measures of velocity, position, and torque^29^. ISOMOVE dynamometer instrument reliability was previously validated for the quadriceps strength measurements^28,30^. The isokinetic dynamometer has been reported to have ICC = 0.088 (0.73–0.94), CV = 7.19, and SEM = 9.98 for isometric knee extensor peak torque measured at 60° knee flexion^31^. Before the pre-intervention evaluation, participants received an introduction to the apparatus. To produce the greatest torque, participants had to sit with their backs supported, hip joints at 90º, and the knee tested at 60° of flexion ^32^. To prevent movement during contraction, straps were placed across the pelvis, midthighs, and chest. At 5.1 cm (2 inches) above the medial malleolus, the shin pad was put in place. The participants were given verbal instructions to keep their arms crossed over their chests and to attain maximum effort of contractions for 5 s. The test included three isometric contractions, with a 2 min rest between the trials. The average score of the three isometric maximum peak torque was taken for baseline measurements.
B. Sprint test (ST): It is a reliable (ICC = 0.95–0.98) and valid test for measuring the performance of speed ^33,34^. For the ST, participants had to sprint with a maximum speed over a 50-m distance. When the participants reached the finishing line, then their performance was measured by measuring the time (in seconds) with a stopwatch (XINJIE, SW8-2008) ^28,35^. Two STs were subsequently performed after 5 min of the recovery period. For the baseline measurement, the lowest of the two scores was taken into account.
C. Single-leg triple hop test (SLTH): In clinical populations, it has been found that the SLTH is both valid and reliable (ICC = 0.97, SEM = 11.17 cm)^36,37^. The SLTH test score was measured using a measuring tape measuring the covered distance in three hops. Participants began the three successive hops with their dominant leg. The distance between the starting point and the back of their heel was measured^30,38,39^. SLTH was performed thrice with a 3 min rest period after every trial. The best score covering the maximum distance was taken as the baseline. The IS, ST, and SLTH were assessed by the same outcome assessor who was blinded to the study.

2. Intervention: The training in PTG and STG was conducted at the same time of the day, i.e., in the evening. Each participant underwent a 15-min standardized warm-up, which included 8–10 min of running, jogging, and stretching exercises for 5–6 min. Interventions in both groups were required to perform three sessions per week on alternate days for eight weeks.

#### Details of training programs in three groups

A. Plyometric training group (PTG): Participants in this group performed three exercises.
i. Bounding: This plyometric exercise uses enormous strides and running-specific action. During these exercises, to cover more distance, there is an increased hip and knee flexion while the arms swing in regular sprinting^40^. The participants performed bounding exercises for 30 m for two repetitions for the first two weeks, and after that, three sets of 30 m for six weeks with a rest period of 3–4 min.
ii. Hurdling: This plyometric exercise uses a 40 cm height total of 8 cones placed in a straight line, 1 m apart, for hurdling while jumping over the cones. The participants had to use both legs to jump over the consecutive cones. For the first two weeks, the participants hurled over eight cones and performed two sets. And for the next six weeks, they performed three sets. The participants were allowed a rest period of 2–3 min after every set.
iii. Drop jumping: This plyometric exercise involved the participants dropping from a height of 40 cm stepper and then jumping forward immediately with maximum effort. For the first two weeks, the participants did drop jumps in two sets of ten repetitions; for the next six weeks, they did three sets. The participants were allowed a rest period of 2–3 min after every set.
B. Strength training group (STG): These exercises were performed bilaterally.
i. Isometric knee extension exercises: Participants sat with their knee flexed at 60^0^ on the Isomove device and were instructed to contract their quadriceps muscle isometrically with maximum force during a 5-s contraction with two minutes of rest between each set of training. Three sets of 10 repetitions were performed three days a week for eight weeks.
ii. Isometric quadriceps exercise: A towel roll was positioned underneath each participant's knee while supine. The participants were told to tighten their thigh muscles as much as possible in order to straighten their knees for 5 s. This exercise consisted of 3 sets of 10 repetitions three days a week for eight weeks.
iii. Straight leg raise (SLR) exercise: Prior to beginning the exercise's lifting phase while lying flat on their backs, participants were told to make a strong isometric quadriceps contraction. The participants were asked to lift their lower limbs 10 degrees with maximal contraction of quadriceps muscles during 5-s holds. This exercise was for three sets of 10 repetitions three days a week for eight weeks.
iv. Isometric hip adduction exercise: The participants were in a crook lying position on the mat and asked to perform isometric contraction of adductors against a soccer ball placed between the knees. Three sets of 10 repetitions (each rep. for 10 s), 10-s rest after each contraction, and 2 min rest after each set, three days a week for eight weeks, were performed^41^.
C. Control group (CG): In this group, participants were not given any specific training; however, they were allowed to continue their regular home training. Their home training included stretching exercises only. They performed a standardized warm-up session of 15 min which included 8–10 min of running, jogging, and stretching exercises for 5–6 min. This warm up session was performed before the baseline and post-intervention measurement only.

Participants in all three groups were allowed to play their football matches once in a week on the night between Thursday and Friday.

3. Post-intervention evaluation: IS, ST, and SLTH were again measured after completing the 8-week training period. These measurements were taken after a gap of 2 days on Sundays following the end of the intervention period.

#### Statistical analysis

Data from 90 participants (30 participants in each group) were analyzed using the SPSS statistical software version 26 (SPSS Inc., Chicago, IL, USA). Baseline values of IS, ST, and SLTH were tested using the Shapiro–Wilk normality test for normal distribution, which revealed no normal distribution for several baseline values. Therefore, non-parametric tests were used for further with-in and between-group analyses. Wilcoxon Signed Ranks Test was used for with-in-group analysis, and Kruskal Wallis Test with post-hoc and Mann–Whitney U tests were used for between-group analyses. p ≤ 0.05 was regarded as significant, and the confidence interval was set at 95%. Effect sizes were calculated using the formula r = z/√n for Wilcoxon Signed Ranks and Mann–Whitney U test. This effect size was interpreted as r less than 0.3—small effect, r between 0.3 and 0.5—medium effect, r greater than 0.5—large effect^42^.

## Results

Data from 90 participants, with 30 participants in each group, were analyzed. Demographic data, descriptive statistics of dependent variables (IS, ST, and SLTH), and Shapiro–Wilk test of normality results are presented in Table 1.

With-in group results: These results are presented in Table 2.

**Table 2:** With-in group results (Wilcoxon signed ranks test) for all three groups.

	PTG			STG			CG		
	z	p-value	Effect size (r)	z	p-value	Effect size (r)	z	p-value	Effect size (r)
IS_Pre–IS_Post	− 4.78	< 0.001*	− 0.61	− 4.78	< 0.001*	− 0.61	− 4.71	< 0.001*	− 0.60
ST_Pre–ST_Post	− 4.78	< 0.001*	− 0.61	− 4.78	< 0.001*	− 0.61	− 4.78	< 0.001*	− 0.61
SLTH_Pre–SLTH_Post	− 4.78	< 0.001*	− 0.61	− 4.70	< 0.001*	− 0.60	− 4.78	< 0.001*	− 0.61

*PTG* plyometric training group, *STG* strength training group, *CG* control group, *IS* isometric strength, *ST* sprint test, *SLTH* single leg triple hop test.

*Significant.

PTG: a significant improvement (p < 0.05) was observed in all three dependent variables (IS, ST, SLTH) with large effect sizes. IS increased by 8.83%. ST decreased by 20.14%. SLTH increased by 7.78%.

STG: a significant improvement (p < 0.05) was observed in all three dependent variables with large effect sizes. IS increased by 15.76%, ST decreased by 30.26%, and SLTH increased by 12.41%.

CG: a significant improvement (p < 0.05) was observed in all three dependent variables with large effect sizes. IS increased by 5.72%, ST decreased by 15.54%, and SLTH increased by 4.41%.

Between-group results: these results are presented in Table 3.

**Table 3:** Between-group (Mann–Whitney U and Kruskal Wallis test) results.

	Between PTG and STG			Between PTG and CG			Between STG and CG			Between all three groups (Kruskal Wallis test)
	z	p-value	Effect size (r)	z	p-value	Effect size (r)	z	p-value	Effect size (r)	p-value
IS_Post	− 5.99	< 0.001*	− 0.77	− 3.51	< 0.001*	− 0.45	− 6.46	< 0.001*	− 0.83	< 0.001*
ST_Post	− 6.14	< 0.001*	− 0.79	− 2.84	0.004*	− 0.36	− 6.49	< 0.001*	− 0.83	< 0.001*
SLTH_Post	− 4.43	< 0.001*	− 0.57	− 3.51	< 0.001*	− 0.45	− 5.93	< 0.001*	− 0.76	< 0.001*

*PTG* plyometric training group, *STG* strength training group, *CG* control group, *IS* isometric strength, *ST* sprint test, *SLTH* single leg triple hop test.

*Significant.

Kruskal Wallis Test: there was a significant (p < 0.001) difference in post-intervention values of IS, ST, and SLTH between all three groups. Tables 4, 5, and 6 show Kruskal Wallis test with post-hoc results for variables IS, ST, and SLTH.

**Table 4:** Kruskal Wallis test with post-hoc results for variable IS.

	Test statistic	Std. error	Std. test statistic	Adj. sig.^#^
CG–PTG	16.833	6.733	2.500	0.037*
CG–STG	50.467	6.733	7.496	0.000*
PTG–STG	− 33.633	6.733	− 4.995	0.000*

*PTG* plyometric training group, *STG* strength training group, *CG* control group, *IS* isometric strength.

^#^Significance values have been adjusted by the Bonferroni correction for multiple tests.

*Significant.

**Table 5:** Kruskal Wallis test with post-hoc results for variable ST.

	Test statistic	Std. error	Std. test statistic	Adj. sig.^#^
STG–PTG	35.917	6.743	5.326	0.000*
STG–CG	− 49.533	6.743	− 7.346	0.000*
PTG–CG	− 13.617	6.743	− 2.019	0.130

*PTG* plyometric training group, *STG* strength training group, *CG* control group, *ST* sprint test.

^#^Significance values have been adjusted by the Bonferroni correction for multiple tests.

*Significant.

**Table 6:** Kruskal Wallis test with post-hoc results for variable SLTH.

	Test statistic	Std. error	Std. test statistic	Adj. sig.^#^
CG–PTG	19.217	6.741	2.851	0.013*
CG–STG	44.633	6.741	6.621	0.000*
PTG–STG	− 25.417	6.741	− 3.771	0.000*

*PTG* plyometric training group, *STG* strength training group, *CG* control group, *SLTH* single leg triple hop test.

^#^Significance values have been adjusted by the Bonferroni correction for multiple tests.

*Significant.

Mann–Whitney U test

PTG and STG: for the post-intervention values of all three variables, there was a significant (p < 0.001) difference between the two groups.

PTG and CG: for the post-intervention values of all three variables, there was a significant (for IS and SLTH, p < 0.001; for ST, p = 0.004) difference between the two groups.

STG and CG: for the post-intervention values of all three variables, there was a significant (p < 0.001) difference between the two groups.

The greatest improvements were observed in STG, followed by PTG and the CG.

## Discussion

The present study aimed to compare the effects of an 8-week-long PT and quadriceps strength training program on the IS of quadriceps, ST, and SLTH in male soccer players. Three groups were created in the present study, 1. STG, 2. PTG, and 3. CG (no intervention). The present study showed significant improvements in the IS of the quadriceps muscle, ST, and SLTH in all three groups. However, the greatest improvements were observed in the STG, followed by the PTG and then the CG. In the present study, the PTG consisted of bounding, hurdling, and drop-jumping exercises. The STG consisted of isometric knee extension exercises at 60°, isometric quadriceps exercise in supine lying, SLR, and isometric hip adduction exercises. The CG did not include any intervention; however, they were allowed to continue their regular home training programs.

Improvements were observed even in the CG that was not given any intervention because participants in this group were allowed to continue their regular home training, which included stretching exercises only. Participants in the CG performed a standardized warm-up session of 15 min which included 8–10 min of running, jogging, and stretching exercises for 5–6 min. This warm-up session was performed before the baseline and post-intervention measurement only. Participants in all three groups were allowed to play their football matches once a week. Therefore, these may be the possible reasons for improvements in CG in IS of the quadriceps, ST, and SLTH.

In the present study, 20–31% improvement was observed in ST in all three groups. The participants in the present study were recruited from university and local soccer clubs. They were not elite or high-performance players; therefore, there was a scope for improvement in ST after training. This might be the reason why such a good improvement was observed.

In agreement with the present study's results, previous research has demonstrated that young adult male athletes' performance and strength can be effectively increased with PT^43–46^. Plyometric exercises have been reported to enhance running performance^6,9^, sprinting^6,7^, leg strength, and muscle power^8^. It can also promote agility^9^. According to Ramirez-Campillo, six-week PT helped 14-year-old soccer players perform better in 20-m sprints^47^. Few studies have reported improved jumping ability following PT^48,49^.

Three phases make up a typical plyometric exercise. The eccentric phase, which is the initial phase, involves quickly stretching the muscles. The amortization phase, the second phase, is a brief period of rest, and the concentric phase, the third phase, is when the athlete makes an explosive muscle-shortening movement^12^. In this way, more force than by a concentric action alone is created by using the muscle's elastic energy that has been stored there^12^. Intending to cut down on time between the eccentric and concentric motions, the athlete quickly repeats this three-part cycle. Athletes become more powerful and faster when the time between eccentric and concentric movement is shortened because nerve, tendon, and muscle functions are improved^50^. Athletes' increased physical power allows them to hit harder, jump higher, and run faster. They also learn specific skills, such as how to protect themselves from injuries^51^. Improvements following the PT may result from neuromuscular adaptations, such as better intermuscular coordination, improved neural drive to the agonist muscles, and modifications to muscle size^11,52^.

Another training program used in the present study was a strength training program that included the isometric exercise of quadriceps, SLR, and adductor strengthening exercises. For the isometric exercise of the quadriceps in the sitting position, the knee angle at 60° of flexion was set, which produces significant torque output (Alonazi et al. 2021). It is reported that lower limb muscle strength improvement could enhance one's capacity for short-duration sprints^53^. Previous research has reported a high correlation between sprinting performance and leg extensor muscle strength (Quadriceps)^53,54^. A recent study has reported that the muscle volume of quadriceps and adductors was larger in sprinters compared to physically active males, which shows the role of quadriceps and adductor muscle strength in sprinting^55^.

## Application to clinical practice

PT involves eccentric exercises; therefore, they may also have disadvantages, particularly at the beginning^12^. Eccentric contractions that are repeated frequently and with high intensity lead to DOMS^22^. The study by Bowers et al.^23^ reported muscle damage after eccentric exercises due to the presence of swelling, DOMS, and reduced peak torque of the muscle. The fall in peak torques immediately after eccentric exercises has been reported in different muscles, like in knee flexor^56^, ankle extensor^57^, and other muscle groups^58,59^. This decline in peak torque following eccentric exercises may be due to both muscle fiber injury and metabolic fatigue^59^. According to Bowers et al.^23^, most muscles, regardless of their regular activity, are likely to show some degree of vulnerability to specific, focused eccentric exercise programs, provided that the exercise is carried out over the appropriate length range. They suggested that, in individuals who are not accustomed to the eccentric exercises, to avoid the debilitating effects of muscle damage and DOMS due to eccentric exercises, a program of eccentric exercises that is vigorous enough to elicit an adaptation response in the muscles but mild enough not to impair the participant seriously should be performed^23^. In the present study, PTG was compared with STG which included isometric quadriceps, adductor and SLR exercises, which are comparatively less strenuous than plyometric exercises. Since more remarkable improvement was found in STG i.e. with the isometric quadriceps, adductor and SLR exercises, therefore, soccer players and coaches can use these exercises instead of plyometric exercises when the intended goal is to improve IS, ST, and SLTH. However, a higher risk of acute hamstring strain injury was found in professional football players with significantly lower isokinetic hamstring strength and a lower hamstring-to-quadriceps strength ratio^60^, therefore, hamstring strengthening exercises should also be performed along with quadriceps strengthening exercises.

## Limitations of the study and scope for future research

In one study, female endurance runners who underwent ten weeks of in-season combined plyometric and resistance training increased their cross-country performance and running economy^61^. Therefore, future research should examine the effect of a combined plyometric and strength training program. In the present study, a stopwatch was used for measuring time for ST, which can vary in time measurement compared to other sophisticated equipment. Therefore, future research can use more sophisticated equipment like photocell equipment for sprint test. Also, the isometric strength of the quadriceps was used in the present study; future studies can use concentric and eccentric strength measurements. The present study recruited soccer players from university and soccer clubs; therefore, they were not elite or high-performance players. The difference in the performance of amateur and elite players has been reported in previous studies favoring elite players^62–65^. A study by Cometti et al.^66^ reported that professional players are different from amateurs in terms of knee flexor muscle strength and short-distance sprinting speed. They also reported that the amateur group's hamstring/quadriceps ratios were much lower than those of the elite group of soccer players. Therefore, before generalizing the results of the present study to elite or high-performance soccer players, future research should be conducted on this population.

## Conclusions

Strength training (consisting of isometric knee extension, isometric quadriceps, SLR, and isometric hip adduction exercises) was found to be more effective than PT (consisting of bounding, hurdling, and drop jumping exercises) in improving muscle strength of quadriceps, sprinting, and lower limb functional performance in the recruited male soccer players. A few adverse effects of PT may occur in players; therefore, strength training can be used instead of PT to improve muscle strength of the quadriceps, sprinting, and lower limb functional performance in non-elite soccer players. However, strengthening of the antagonist muscle, i.e., the hamstring, should also be performed along with quadriceps strengthening, as suggested in previous studies, to prevent hamstring injuries.

## Data Availability

The data associated with the paper are not publicly available but are available from the corresponding author on reasonable request.
